# Can We Increase the Drought Tolerance of Perennial Ryegrass (*Lolium perenne* L.) to Preserve Grassland Ecosystem Services? A Case Study with Three Bulgarian Varieties

**DOI:** 10.3390/plants14233704

**Published:** 2025-12-04

**Authors:** Aneliya Katova, Plamen Marinov-Serafimov, Irena Golubinova, Bogdan Nikolov, Slaveya Petrova

**Affiliations:** 1 Institute of Forage Crops, Agricultural Academy, St. “Gen. Vladimir Vazov” 89, 5800 Pleven, Bulgaria; aneliya.katova@ifc-pleven.org; 2 Institute of Ornamental and Medicinal Plants, Agricultural Academy, St. “Ezerata”, 1222 Sofia, Bulgaria; plamen.serafimov@idlr.bg (P.M.-S.); irena.golubinova@idlr.bg (I.G.); 3 Department of Ecology and Environmental Conservation, Faculty of Biology, University of Plovdiv “Paisii Hilendarski”, 24 Tsar Asen Str., 4000 Plovdiv, Bulgaria; nikolov81bg@uni-plovdiv.bg; 4 Department of Microbiology and Environmental Biotechnologies, Faculty of Plant Protection and Agroecology, Agricultural University, 12 Mendeleev Blvd., 4000 Plovdiv, Bulgaria

**Keywords:** climate change, water scarcity, osmotic stress, forage production, genetic breeding, tetraploid, gene donors

## Abstract

*Lolium perenne* L. (perennial ryegrass) has various applications, including as a high-quality forage species for livestock feed; in seed mixtures used for revegetation of eroded or degraded areas as well as for lawns due to its resistance and rapid germination; for erosion control on slopes and areas with excessive steepness; for phytoremediation of soils contaminated with potentially toxic elements due to its ability to accumulate metals in its tissues; and as a cover crop to improve soil conditions and control erosion. Accordingly, *L. perenne* provides several ecosystem services, primarily related to soil stability, agriculture, and recreation. Climate change poses challenges for *L. perenne*, particularly heat and drought stress, which can reduce its yield and alter its geographical distribution. Climate change also impacts the interactions between *L. perenne* and its environment, affecting aspects like phenology (e.g., flowering time), carbon fixation, and overall resilience. However, the species’ significant genetic and endophyte-related variability may allow for adaptation. The aim of the present study was to assess the drought tolerance of three Bulgarian varieties of *L. perenne*, namely Harmoniya (diploid), Tetrany, and Tetramis (tetraploids). We performed induced drought stress under laboratory conditions and monitored its effect on plants in the early stages of growth and development. A variety-specific response was found regarding the effect of different concentrations of sucrose on seed germination, primary root and stem elongation (cm), fresh biomass accumulation (g), as well as on seedling vigor index and plant development. Field experiments and yield elements were also used to assess drought susceptibility and sensitivity to stress in a real environment. The tetraploid perennial ryegrass varieties Tetrany and Tetramis showed better germination, growth, and development in laboratory tests and had higher and more stable field productivity under both optimal and stress conditions than the diploid variety Harmoniya. Ploidy was the factor that characterize them as drought-tolerant genotypes under water-limited conditions, and its potential could be used in future breeding programs.

## 1. Introduction

Plant growth and productivity are influenced by many biotic and abiotic factors. The impacts of global climate change pose a major challenge to the sustainability and productivity of grass species [1]. Global warming trends suggest an increase in atmospheric temperature, as well as in the frequency and duration of periods of high temperatures, drought, and floods. Water scarcity is the main factor hampering world agriculture by limiting crop productivity, and global climate change is increasing the frequency of severe drought conditions [2,3]. Plant abiotic stress, such as drought stress, threatens global food availability as the world’s population and per capita food consumption increase. Drought severely affects plant growth and development with significant reductions in crop growth rates and biomass accumulation as it disrupts basic metabolic, physiological, and biochemical processes [4,5]. Understanding the physiological, biochemical, and ecological effects associated with these stresses is crucial for better management of agroecosystems [6,7,8]. Drought, high salinity, and extreme temperatures are major adverse environmental stresses that plants often encounter, which is further complicated by the potential impact of climate change [8,9,10,11,12]. In the face of a warming climate and reduced water resources, it is clear that searching for any factors that decrease water use is strongly recommended. Cool-season turf grasses often suffer from extended periods of drought during the summer, but water supplies utilized to irrigate turf are limited and are in competition for use by agriculture, recreation, and others [11,12,13,14,15,16].

Grasslands are considered to be among the most species-rich ecosystems in Europe, providing multiple habitats and supporting high levels of biodiversity, no matter if they occur as natural stands or as grazed meadows and pastures [3,6,9,16,17,18]. Perennial ryegrass (*Lolium perenne* L.) is the most widely grown temperate grass species globally. It is known as a high-quality forage species for livestock feed (grazing, hay, and silage), and it is preferred by farmers because it is easy to establish, tolerant to intensive grazing, trampling, and frequent mowing, has an excellent nitrogen uptake and, most importantly, a higher nutritional value in comparison with perennial grasses [6,7,8]. *Lolium perenne* is a common species in seed mixtures for revegetation of eroded or degraded areas as well as for lawns, parks, and gardens due to its resistance and rapid germination [11,18,19,20]. As a part of the landscape, it protects soil from water and wind erosion, especially on slopes and areas with excessive steepness, and enriches it with organic substances and maintains and improves its fertility [20,21]. It is widely used for phytoremediation of soils contaminated with potentially toxic elements, thanks to its ability to accumulate metals in its tissues, and as a cover crop to improve soil conditions and control erosion [22]. Accordingly, *L. perenne* provides several ecosystem services, including provisioning, regulating, and supporting, which are primarily related to soil stability, agriculture, and land reclamation. Its root systems help prevent soil erosion, bind soil particles together, and improve soil structure. As a forage crop, it supports livestock production, and its use as turf grass provides economic and recreational benefits.

Climate change poses challenges for *L. perenne*, particularly through heat and drought stress, which can reduce its yield and alter its geographical distribution. Climate change also impacts the interactions between *L. perenne* and its environment, affecting aspects like phenology (e.g., flowering time), carbon fixation, and overall resilience. The productivity of perennial ryegrass, which is among the most widespread grass species in temperate zones, can be compromised by drought because, unlike many grass species, it forms a relatively shallow root system, which limits drought tolerance [17,23,24,25]. As irrigation is usually not economically justified for grassland, there is a need to grow more drought-tolerant varieties unless the species is replaced by more drought-tolerant species. However, the species’ significant genetic and endophyte-related variability may allow for adaptation over time. A review of the scientific research related to drought tolerance in *L. perenne* (1990–2024) revealed that they were predominantly focused on phenotypic changes, response mechanisms, and techniques to enhance its drought and heat resistance, concluding that there is a need to strengthen research on the functions and mechanisms of genes related to drought stress [17].

As its description as a perennial grass suggests, it is understood that ryegrass completes its ontogenetic development not in one, but in many (3–4 and more) years. Unlike annuals, where the life cycle is completed in less than one calendar year, perennial ryegrass is exposed to environmental factors year-round for many years, with frequent stress situations [26]. Drought stress leads to decreased forage quality and biomass synthesis, combined with premature leaf yellowing and senescence [17]. Temperatures over 38 °C in the summer could lead to accelerated metabolic and physiological changes [17]. These environmental stresses exceed the optimal growth conditions for perennial ryegrass, resulting in decreased forage yield and quality. This is why the resistance of the population to adverse factors of the local ecological conditions is of great importance for the productivity and longevity of the grassland [27,28,29].

World plant breeding has created many varieties of perennial ryegrass with specific eco-adaptability. In the world, the first breeding activity with perennial ryegrass began in 1889 at the Welsh Experimental Station (Great Britain) [30]. The OECD World Trade List for 2024 includes 1775 varieties of perennial ryegrass [31]. The selection of the most locally suitable perennial ryegrass is carried out in many countries, focusing upon different ecological features. Morphological characteristics, biological properties, and economic qualities are improved. The longevity and productivity of perennial ryegrass varieties depend on their genetic backgrounds and the complex impact of environmental conditions and resources (including diseases). The most long-lasting varieties are those created in the regions of the genetic origin of the species [6,7,8,14,15,16].

Bulgaria is located on the border between the Mediterranean and Caucasian gene centers, where the species originated and where the diversity is greatest. Local populations and varieties of perennial ryegrass are more winter-resistant and long-lasting than all introduced kinds in the environmental conditions of Bulgaria [32]. Development of new crops, interspecific gene transfer, and the origin of new crops can be traced with the help of polyploidy breeding [33]. There is a correlation between polyploidy and perennialism [34]. Polyploids appear different from their progenitors in morphological, ecological, physiological, and cytological characteristics that can contribute both to exploitation of a new niche and to reproductive isolation. Therefore, polyploidy is a major mechanism for adaptation and speciation in plants.

Based on the above, our aim was to study the drought tolerance of three Bulgarian *L. perenne* varieties—Harmoniya (diploid), Tetrany, and Tetramis (tetraploids)—as follows: (1) assessment of the effect of induced drought in lab conditions; (2) assessment of the effect of drought in field conditions; and (3) comparing plant productivity and their sensitivity to drought stress.

## 2. Materials and Methods

### 2.1. Lolium perenne Varieties

Three varieties of *L. perenne*, created by different methods (Table 1) at the Institute of Forage Crops—Pleven (Bulgaria), were used in the drought tolerance testing.

Harmoniya is the first Bulgarian diploid variety of perennial ryegrass, registered the country’s variety list in 2010. It was created by individual phenotypic selection and is a polycross of 91 elite diploid genotypes of native origin [35].

Tetrany and Tetramis, two new tetraploid perennial ryegrass varieties, were recognized by the Executive Agency for Variety Testing, Approbation and Seed Control (EAVTASC, a Bulgarian government agency) in 2017 [36,37]. They were created by induced polyploidy of the local breeding population, flow cytometric screening, and phenotypic selection of tetraploids followed by polycross (multiple hybridization) of 45 elite genotypes (for Tetramis) or 52 elite genotypes (for Tetrany). After this, there was continuous reproduction to the C 4 generation.

**Table 1:** Bulgarian varieties of perennial ryegrass *Lolium perenne* (L.) used in the study.

Variety	Ploidy Level	Methods of Creation	Reference
Tetramis	4n	Polyploidization, flow cytometric screening, selection, polycross	[37]
Tetrany	4n	Polyploidization, flow cytometric screening, selection, polycross	[36]
Harmoniya	2n	Selection, hybridization, polycross	[35]

### 2.2. Laboratory Test for Induced Drought

The experiment was carried out in laboratory conditions, and the effects of two factors were investigated:

Factor A—*Lolium perenne* L. varieties, with three options: a1—Tetrany; a2—Tetramis; a3—Harmoniya;

Factor B—sucrose concentration, with five experimental doses: b0—0.0% (control); b1—1.0% sucrose; b2—3.0% sucrose; b3—6.0% sucrose; b4—9.0% sucrose, and b5—12% sucrose.

All drought stress models are based on the reduction in the available water for plants, which can be achieved by limiting irrigation or by applying osmotic stress as a simulation of drought [38,39]. The second approach is related to an increase in the osmotic pressure of the growing media in comparison with that of plant tissues. This process simulates the soil conditions when the concentration of solutes grow as a result of the decrease in water content. Different compounds are used in such experiments, and polyethylene-glycol (PEG), mannitol, sorbitol, and sucrose are widely applied. Each compound has its own pros and cons according to the existing literature [38,39,40]. Sucrose was chosen for these experiments, as it is reported as a very good agent to induce osmotic stress in plants when present at a high concentration in the medium. Such concentrations of external sucrose cause water to leave the cells, thus lowering the water potential [40,41,42,43].

The drought tolerance test was set as follows: 20 seeds of perennial ryegrass per variety (according to factor A) were placed between two layers of filter paper Filtrak 388 in 90 mm Petri dishes, and 5 mL of sucrose solution were pipetted onto the top filter paper (sucrose concentration according to Factor B). Each variant consisted of six repetitions; thus, a total of 90 Petri dishes were prepared and placed in a thermal cabinet for 48 h at 23 ± 2 °C. Dark conditions were employed for a 14-day period.

### 2.3. Assessment of Effect of Drought Under Laboratory Conditions

To evaluate the influence of water deficit on studied *L. perenne* varieties, the following biometric indicators were used: number of germinated seed; root and stem length, cm; and fresh biomass weight, g. Seeds were counted as germinated when the root length was more than ≥3.0 mm. Length was measured using graph paper, and biomass was weighted on an analytical balance.

The drought effect on the experimental plants was assessed based on the set of selected indicators as explained below.

Germination percentage (*GP*) of tested seed per variant was calculated using the formula proposed by Bewley et al. [44]:

$$GP\%=\left(\frac{Number\;of\;germinated\;seeds}{Total\;number\;of\;seeds\;tested}\right)\times 100$$

Germination percentage relative to control (*GPC%*) was calculated according to Ibrahim et al. [45]:

$$GPC\%=\left(\frac{Germination\;of\;the\;treated\;group\;}{Germination\;of\;the\;control\;group\;}\right)\times 100$$

where

Germination of the treated group is the percentage of seeds that germinated under the treated condition;

Germination of the control group is the percentage of seeds that germinated in the control variant.

The inhibition rate (*IR*) was determined by equation of Ahn et al. [46]:

$$IR\%=\left(\frac{C-T}{C}\right)\times 100$$

where

*C*—parameter in the control variant;

*T*—parameter for the variant.

Positive “+” values indicate an inhibitory effect, while negative “−” values indicate a stimulatory effect.

The effective concentration that inhibits germination in 50% of the seeds (*EC50*) was calculated according to the *IR* as the percentage of germinated seeds. Calculating *EC50* involved fitting experimental data from dose–response experiments into a nonlinear regression model using logistic functions. Once the data were plotted, the concentration corresponding to the midpoint of the maximum response was determined and reported as the *EC50* [47].

Seedling vigor index (*SVI*) was calculated using the equation of Abdul-Baki & Anderson [48]:

$$SVI=\left(\frac{{LS}_{cm\;}\times {GP}_{\%}}{100}\right)$$

where

*LS*_cm_—seedling lenght (cm);

*GP_%_*—germination percentage.

Growth Index (*GI*) was also calculated following the formula of Gariglio [49]:

$$GI=\left[\left(\frac{G}{G_{0}}\right)\times \left(\frac{L}{L_{0}}\right)\right]\times 100$$

where

*G* is the percentage of germinated seeds in the tested sucrose concentrations;

*G_0_* is the percentage of germinated seeds into the control;

*L* is the length of seedling in the experimental variants, %;

*L_0_*—length of the seedling in the control, assumed as 100%.

### 2.4. Assessment of the Drought Effect in Field Conditions

Ryegrass seeds of three Bulgarian varieties were sowed on the field of Institute of Forage Crops—Pleven in the middle of May 2021 following the standardized agricultural procedure for perennial grasses [50]. The sowing of the seeds was made in rows, with a row spacing of 36 cm; the number of rows was 40, on a plot measuring 14.0 m × 5.00 m = 70 m^2^, per variety. All experiments were carried out in triplicate following spatial randomization of plots. Plant fertilization was carried out annually during the growing season (spring and autumn) with 60 kg N kg ha^1^ in the form of ammonium nitrate (NH_4_NO_3_). The experiments were carried out under non-irrigated conditions. Seed productivity and plant development were recorded two years later (2022–2023), since in the first year of establishment (2021) the plants only reach vegetative development.

The limiting environmental factors were favorable and unfavorable years in terms of the meteorological elements, as the soil properties and all other factors were the same in all plots. The average annual temperatures for the study period (2022 and 2023) were 13.7 °C for 2022 and 14.5 °C for 2023, while the annual amount of precipitation was smaller in 2022 (386.1 mm) and bigger in 2023 (530.0 mm).

The Aridity Index proposed by de Martonne [51] was used to assess the environmental conditions. It represents the ratio between the annual amount of precipitation and the average annual temperature +10 °C. For 2022, the index value was 16.29 (semiarid climate), and for 2023 it was 21.6 (moderately dry climate); thus, 2022 emerged as the drier year for the two-year period of the study.

The set of selected drought tolerance indices [52] were used to assess the drought effect on the three *L. perenne* varieties in field conditions as described below.

The Drought Tolerance Index (*DTI*) evaluates whether a genotype maintains productivity under drought conditions and was calculated according to the formula proposed by Lan [53]:

$$DTI=\frac{\left(Y_{s}\times \left(\frac{Y_{s}}{Y_{p}}\right)\right)}{\bar{Y_{s}}}$$

Drought Susceptibility Index (*DSI*), which measures how strongly a variety reacts to drought compared to others, was calculated according to Fischer and Maurer [54]:

$$DSI=\;\frac{1-\left(Y_{s}/Y_{p}\right)}{1-\left({\bar{Y}}_{s}/{\bar{Y}}_{p}\right)}$$

Percentage Sensitivity to Stress Index (*SSPI*) measures the percentage of yield loss of a genotype under stress relative to its performance under optimal conditions and was proposed by Moosavi et al. [55]:

$$SSPI=\frac{Y_{p}-Y_{s}}{2{\bar{Y}}_{p}}$$

Tolerance Index (*TOL*) was proposed by Rosielle & Hamblin [56] to measure the absolute yield loss in the presence of stress:

$$TOL=Y_{p}-Y_{s}$$

Yield Index (*YI*) evaluates the relative productivity of a variety under adverse conditions and was introduced by Gavuzzi et al. [57]:

$$YI=\frac{Y_{s}}{{\bar{Y}}_{s}}$$

where

*Y_p_*—potential yield of each individual genotype under non-stress conditions;

Y_s_—yield of each individual genotype under stress conditions;

*Ȳ_p_*—average potential yield of all genotypes under non-stress conditions;

*Ȳ_s_*—average yield of all genotypes under stress conditions.

The yields recorded in 2023, characterized as moderately wet and very warm in terms of temperature and precipitation conditions, were considered as the potential yield—*Yp*, while the yields recorded in 2022 (a dry and moderately warm year) were designated as *Ys—the yield under stress conditions.*

### 2.5. Statistical Analyses

The results were processed mathematically and statistically utilizing STATGRAPHICS Plus for Windows, Version 2.1. The values presented in the figures and tables are the mean ± standard deviation. Comparisons between all treatments and the control were made using Tukey’s post hoc test. Different letters indicate significant differences at the *p* < 0.05 level.

## 3. Results

### 3.1. Effect of Induced Drought on Seed Germination

The results (Table 2) indicate that the seed germination in the experimental variants decreased with increasing sucrose concentrations (relative to Factor B). Germination percentage (*GP*) varied from 2.5% to 87.5% for Tetrany, from 2.5% to 85.0% for Tetramis, and from 2.5% to 72.5% for Harmoniya. All of the applied concentrations of sucrose (from 1.0 to 12.0%) had an inhibitory effect on seed germination in all of the *L. perenne* varieties when compared to the control (0.0%). The weakest negative effect of the 1.0% sucrose solution was found for Tetrany (*GPC* = 91.4%), followed by Tetramis (*GPC* = 82.4%), while the strongest effect of the same solution occurred in Harmoniya (*GPC* = 51.7%). The inhibition rate for the Tetramis variety increased in the 3% sucrose solution up to 70.6%, and after that the trend was slightly upward. The inhibition rate for the Tetrany variety also demonstrated a significant increase from 8.6% (1.0% solution) to 57.1% (3.0% solution) and to 80.0% (6.0% solution), continuously increasing to 97.1% at 12.0% solution. The Harmoniya variety exhibited a different inhibition rate, which started from 48.3% (1.0% solution), increased to 62.1% (3.0% solution), and rose to 96.6% in all other sucrose concentrations (6.0–12.0%). Our results correspond well with the findings of Cyriac et al. [58] and Waldron et al. [59], who reported that the enhancement of both osmotic and salt stresses significantly reduced seed germination in perennial ryegrass.

**Figure 1:**
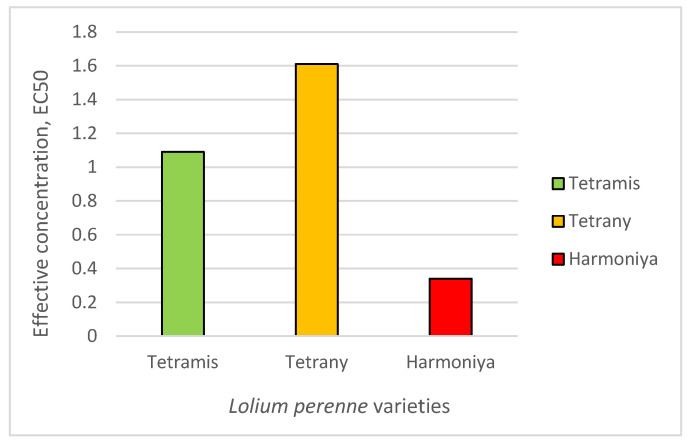
Effective concentration of sucrose that inhibits the germination of 50% of the seeds (*EC50*).

The effective concentration that inhibits the germination in 50% of the seeds (*EC50*) ranged from 0.34 to 3.21% sucrose (Figure 1), and the studied *L. perenne* varieties could be arranged in the following ascending order: variety Harmoniya [average *EC50* = 0.78 (0.34–1.76)] < variety Tetramis [average *EC50* = 1.76 (1.09–2.83)] < variety Tetrany [average *EC50* = 2.27 (1.61–3.21)]. Differences in the *EC50* values can be explained by genetic differences and the way the varieties have been produced, since the comparisons were made under the same controlled laboratory conditions.

### 3.2. Effect of Induced Drought on Plant Growth and Development

Analogous to seed germination, different concentrations of sucrose inhibited primary root and stem growth and development in all of the *L. perenne* varieties (Table 3). Differences in plant development at varying levels of osmotic stress are an indicator of the degree of drought tolerance.

The effect of sucrose-induced drought stress on primary root length (cm) was lowest at the 1.0% concentration where the *IR* varied from 23.7% (Harmoniya) to 29.1% (Tetramis). When the concentration rose to 3.0%, the inhibition rate sharply reached 93.9% (Tetramis) to 98.7% (Harmoniya), continuously increasing to 100.0% at higher doses of sucrose. These results demonstrated that all perennial ryegrass varieties experienced significant physiological stress at the earliest stages of development (BBCH 00-09), despite the reported drought resistance of the species [27,28,29].

The length of the shoot, ranging between 0.05 and 8.90 cm, was comparatively greater than that of the root (between 0.00 and 7.15 cm). Primary shoot length was greatest at the lowest applied sucrose concentration of 1.0% where *IR* ranged from 22.0% (Tetrany) to 41.2% (Harmoniya). The highest degree of inhibition (*IR* from 98.3 to 99.4%) was found at the highest sucrose concentration of 12%. Specific genotypic differences in the response of individual varieties have also been reported. In the variety Harmoniya, the length of the primary shoot was the lowest on average for all variants of the experiment (2.34 cm), while in variety Tetrany it was the highest (3.35 cm).

In terms of the whole seedling length, Tetrany had the best performance (0.15–16.05 cm), followed by Tetramis (0.1–15.1 cm), while Harmoniya demonstrated significantly weaker development (0.05–12.53 cm) even without induced stress (in the control). This was the variety that was found to be most susceptible to sucrose impact as the *IR* was at about 30% greater than in the two tetraploid varieties.

### 3.3. Effect of Induced Drought on Biomass Accumulation

**Figure 2:**
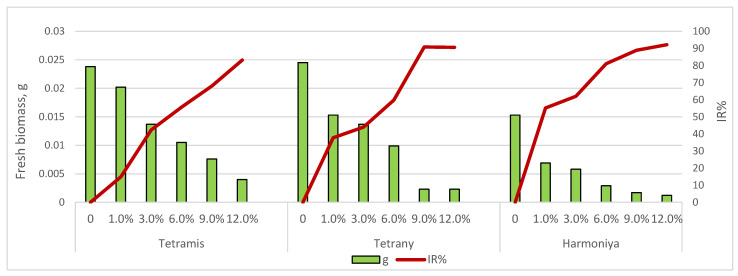
Effect of sucrose concentration on fresh biomass accumulation of the seedlings.

The dynamics of accumulation of fresh biomass of germs in the early stages of growth depended on the variety, and the applied concentration of sucrose is presented in Figure 2. Drought stress in terms of shoot weight caused by the application of the lowest sucrose concentration (1.0%) had a relatively lower suppressive effect in variety Tetrany (*IR*% = 14.89) and a greater effect in variety Harmoniya (*IR*% = 55.12). A greater depression in the accumulation of fresh germ biomass was reported with increasing sucrose concentration (from 3 to 12%), the differences being statistically significant (*p* = 0.05). The greatest inhibitory effect was observed at the highest sucrose concentration (12.0%) in all ryegrass varieties (IR% ranged from 83.16 to 92.16). A reduction in primary shoot fresh biomass is a common response of crop plants when subjected to water deficit [27,28,29].

**Figure 3:**
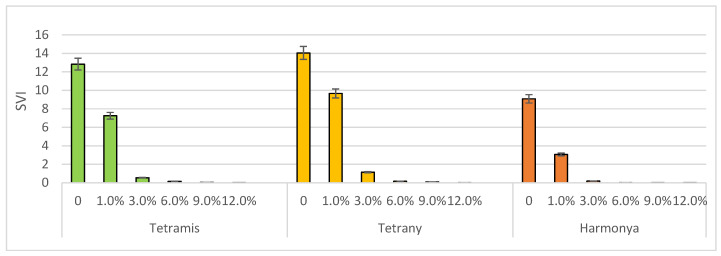
Effect of sucrose concentration on seedling vigor index (*SVI*). The bars represent the +/− standard error of confidence interval of arithmetic mean.

Seed vigor represents a single concept that reflects numerous characteristics and was proposed to determine the seed quality and to uniform the emergence potential of plants in the field under a variable range of environments [48]. It is influenced by multiple factors in real agricultural conditions, i.e., species, genotype, and environmental properties, but it is widely used in controlled laboratory experimental conditions. Two tetraploid varieties had better *SVI* values both in the control and in the induced drought variants when compared to the diploid variety Harmoniya. Tetrany was found have the most vigor, as its *SVI* values ranged from 14 to 0.004 (Figure 3).

**Figure 4:**
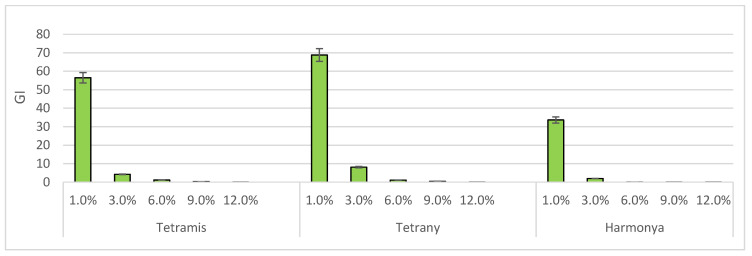
Effect of sucrose concentration on Growth Index (*GI*). The bars represent the +/− standard error of confidence interval of arithmetic mean.

Another useful complex index, the Growth Index [49], was also calculated to better evaluate the effect of the induced drought on plants. It can be seen from Figure 4 that the two tetraploid varieties have again been proven to be more tolerant to harmful conditions than the diploid one.

### 3.4. Drought Effect Evaluation in Field Conditions

Field experiments were conducted to assess the drought tolerance in the real environment as well as to allow for the assessment of the effect of yield. Seed yields for the three studied *L. perenne* varieties were the highest in the first harvest year, 2022, with an average value of 830.56 kg ha^−1^, and the ranking in descending order was Tetramis (1200.78 kg ha^−1^) > Tetrany (890.29 kg ha^−1^) > Harmoniya (400.63 kg ha^−1^). In 2023, the average seed yield was 420.56 kg ha^−1^, and the ranking in descending order was Tetrany (560.52 kg ha^−1^) > Tetramis (480.75 kg ha^−1^) > Harmoniya (220.40 kg ha^−1^) (Table 4). Correspondingly, the average height of the plants was as follows: Tetramis (85.93 cm) > Tetrany (82.28 cm) > Harmoniya (75.05 cm).

According to the Drought Tolerance Index (*DTI*), which was used for assessing the potential of the studied varieties to maintain productivity under drought conditions, Tetramis had the best adaptation having two times greater value than Tetrany and four times greater value than Harmoniya (Table 4). The Drought Susceptibility Index (*DSI*) was introduced to measure how strongly a variety reacts to drought compared to others. According to the literature, when *DSI* values are less than one, the variety can be considered drought-tolerant; thus, all three studied *L. perenne* varieties fit well in this group. Two of the indices calculated, Percentage Sensitivity to Stress Index (*SSPI*) and Tolerance Index (*TOL*), were applied to measure the yield loss under drought conditions. Lower values indicating reduced sensitivity to stress were determined for Harmoniya and Tetrany, while Tetramis was the most affected. The Yield Index (*YI*) indicates the relative productivity of a variety under adverse conditions, and higher values indicate greater tolerance to water deficit stress. Thus, the two tetraploids had more stable development and higher drought tolerance than the diploid variety.

### 3.5. Drought Tolerance Assessment

Analyzing the obtained results from both the laboratory and field experiments, it can be concluded that varieties Tetrany and Tetramis have the potential to be more resistant to drought stress, while variety Harmoniya can be identified as susceptible to drought stress.

Through dispersion analysis, the hierarchical distribution of factors and their weight on the manifestation of drought stress affecting the studied indicators (seed germination, root and shoot length, and seedling biomass) was determined (Table 5 and Table 6). The variance analysis showed that the dynamics of the studied indicators depended to a lesser extent on factor A (genotype) as η^2^ was 4.4 for seed germination process (Table 5) and around 1.0 for plant growth (Table 6) Factor B, sucrose concentration, simulating the drought conditions, had a significantly higher share of the total variation and the strongest influence on the dynamics of the studied indicators. The η^2^ value was 90.3 for seed germination and ranged from 72.9 to 83.4 in relation to plant growth. The mean values for all factors were lower (*p* = 0.05) in the Harmoniya variety (variable a3).

A significant negative correlation (r) was found (Table 7) between the sucrose concentration applied and various seed laboratory germination parameters. Specifically, the correlations were as follows: germination (r values ranging from −0.927 to −0.895), root length (r from −0.774 to −0.792), shoot length (r from −0.787 to −0.880), seedling length (r from –0.785 to –0.842), and the fresh biomass produced per individual seedling (r from –0.835 to –0.966). These results clearly indicate that increasing sucrose concentration has a distinctly inhibitory effect on the early morphological development of the perennial ryegrass cultivars examined in the study across all analyzed morphometric parameters.

**Figure 5:**
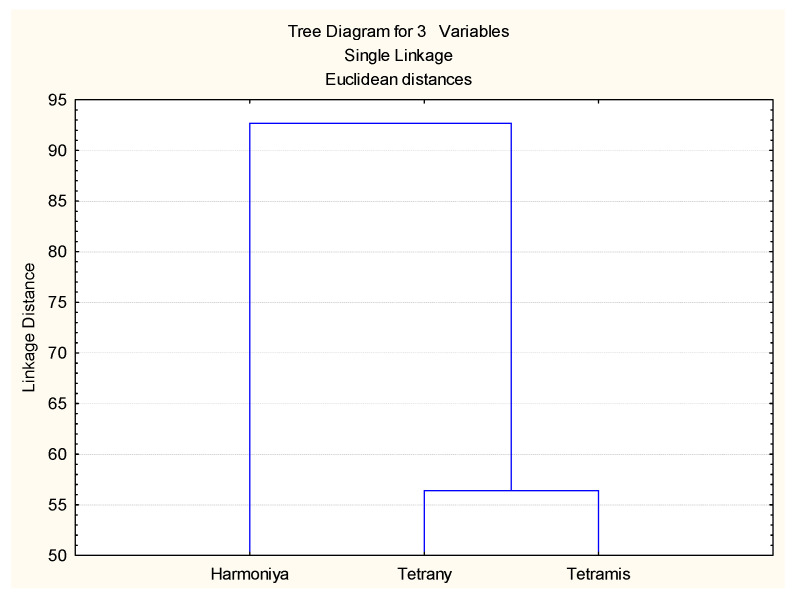
Cluster analysis of the *L. perenne* varieties based on drought tolerance indices.

Cluster analysis was performed based on the values of all indices used, and the results are presented in Figure 5. The two tetraploid varieties (Tetrany and Tetramis) were closely linked in a cluster, while the third variety Harmoniya (diploid) was quite different in its response to drought stress in both the laboratory and the field experiments.

## 4. Discussion

*Lolium perenne* is recognized as one of the most important spontaneous and cultivated forage species worldwide, especially in temperate climates, due to its stability to trampling and grazing, as well the climate-change-induced decline of traditional agricultural practices [60,61,62]. At present, the arid climate poses significant issues to permanent grasslands, and *L. perenne* has a specific value as there is substantive evidence that the ecosystems where it is dominant are more resistant to lower precipitation and temperature increase [20]. Grazing can significantly disrupt the structure and functioning of grassland ecosystems as it directly reduces shoot biomass, thus altering the photosynthetic rates and carbon fixation [63]. As *L. perenne* has a dense root system, it contributes to keeping the soil stable and preventing erosion, as well as diminishing the leaching of nutrients. From an economic point of view, pastures are the cheapest forage, but the key is the availability of a sufficient amount of biomass with high digestibility and nutritional value. The multi-cutting and perennialism of *L. perenne* are important prerequisites for the formation of the total biomass production for the entire period of use of the grassland. It is subject to annual and multi-year influence of environmental factors, with frequent stressful situations. Resistance to the adverse factors of local environmental conditions is of great importance for its productivity and longevity. So, the studies should consider the risk of the potential disappearance of *L. perenne* from the ecosystems in some regions due to the unfavorable climatic changes and its replacements by other species. This would affect the ecological balance in the grassland ecosystems, provoking different natural succession series, and could decrease their performance and functions. This problem can be addressed by breeding programs aiming to develop new perennial grass varieties with high forage and seed productivity, high forage quality and high adaptive potential for pasture, and hay and landscape improvement use in the context of drought increments [64,65]. One of the most critical choices to be made in breeding for drought tolerance is that of the selected environment. Genes for drought tolerance are found in populations occupying frequently arid habitats. Donors of drought stress tolerance should be sought not in well-maintained pastures (fertilized, irrigated, and maintained habitats) but in places with extreme conditions that limit the spread of the species where the genes have already been ecologically tested in evolution. The evaluation of cultivars and germplasm from different geographic regions can be useful for discovering tolerant genotypes for drought resistance in the early growth phases. This necessitates the study and use of rapid and rational methods for evaluating specimens during the breeding process, and those potentially resistant to induced drought resistance will be used in future breeding programs to create drought-resistant genotypes. There are no biological barriers to increasing the adaptive and productive potential through hybridization of the ecologically stable naturally growing populations and the newly created productive varieties and subsequent polyploidization [32,35,36,37].

In the present study, we have compared the drought resistance of three Bulgarian varieties of *L. perenne*—one diploid (Harmoniya) and two tetraploids (Tetrany and Tetramis). As a result of the comparative testing of Bulgarian and foreign varieties of perennial ryegrass, it was established that tetraploids show the highest resistance and productivity in the conditions of the Central Balkan region (Bulgaria). In the foothills of Bulgaria, Tetramis and Tetrany have the best adaptability, resistance, and productivity [32,35]. Tetraploids have been found to have many other positive qualities, including better foliage, taller plants, higher forage and seed productivity, higher water and sugar content, higher digestibility than diploids, faster germination, greater mass per 1000 seeds, better palatability, better disease resistance, winter hardiness, and higher nutritional value; ruminants have a preference for them, and their creation is considered a major advance in perennial ryegrass breeding [50]. Furthermore, the findings of the present study identify their potential in terms of drought stress tolerance. A variety-specific response was found regarding the effect of different concentrations of sucrose on seed germination, primary root and stem elongation (cm), fresh biomass accumulation (g), as well as on seedling vigor index and plant development. The ability of perennial ryegrass seeds to germinate in high drought stress conditions was reported in many studies, along with their ability to tolerate salinity levels up to 200 mM NaCl concentration and ph in the range between 3 and 11 [66]. Other authors revealed that perennial ryegrass resists stress by forming smaller and thicker leaves and expanding the total surface area of its roots to absorb more water [17]. Our findings correlate well with these data as the inhibition rate was more pronounced in the shoot length than to the roots (Table 2). By reducing the aboveground biomass, plants can decrease water consumption and evaporation while, correspondingly, the increased underground biomass helps roots absorb water more effectively, thereby enhancing plant adaptability to drought stress. These intraspecific differences can be used in breeding programs for the development of drought-tolerant perennial ryegrass cultivars [58,59].

## 5. Conclusions

Although it is resilient to environmental stress, *L. perenne* is considered to be susceptible to climate change, especially in terms of aridity. To address this issue, we assessed the drought tolerance of three Bulgarian varieties—two tetraploid (Tetrany and Tetramis) and one diploid (Harmoniya)—in both laboratory and field experiments. Ploidy level was found to be the main factor contributing to an increased drought resilience, and tetraploids demonstrated better growth and development, as well as higher and more stable field yield in drought stress conditions. Our study confirmed that the ploidy manipulation method could be used as an efficient breeding system to achieve new desirable characteristics with significant tolerance to abiotic stresses. The results obtained not only provide guidance for future research on perennial ryegrass under drought stress but also offer valuable information for breeding programs and further studies exploring molecular response mechanisms.

## Figures and Tables

**Table 2:** Effect of induced drought on the seed germination in the three *Lolium perenne* varieties. The means showing different letters are significantly different according to Tukey’s post hoc test (*p* < 0.05).

		Factor A Genotype								
		Tetramis			Tetrany			Harmoniya		
Indicators		*GP*, %	*GPC*, %	*IR*, %	*GP*, %	*GPC*, %	*IR*, %	*GP*, %	*GPC*, %	*IR*, %
Factor B Sucrose concentration, %	0.0%	85.0 e	100.0	0.0	87.5 f	100.0	0.0	72.5 d	100.0	0.0
	1.0%	70.0 d	82.4	17.6	80.0 e	91.4	8.6	37.5 c	51.7	48.3
	3.0%	25.0 c	29.4	70.6	37.5 d	42.9	57.1	27.5 b	37.9	62.1
	6.0%	22.5 c	26.5	73.5	17.5 c	20.0	80.0	2.5 a	3.4	96.6
	9.0%	12.5 b	14.7	85.3	10.0 b	11.4	88.6	2.5 a	3.4	96.6
	12.0%	2.5 a	2.9	97.1	2.5 a	2.9	97.1	2.5 a	3.4	96.6

**Table 3:** Effect of induced drought on root and shoot length of the studied ryegrass varieties. The means showing different letters are significantly different according to Tukey’s post hoc test (*p* < 0.05).

Factor A Genotype Variety	Factor B Sucrose Concentration, %	Root		Shoot		Seedling	
		Length, cm	*IR*, %	Length, cm	*IR*, %	Length, cm	*IR*, %
Tetramis	0.0%	6.45 c	0.0	8.65 d	0.0	15.1 c	0.00
	1.0%	4.57 b	29.1	5.79 c	33.1	10.6 b	31.41
	3.0%	0.40 a	93.9	1.77 b	79.5	2.17 a	85.65
	6.0%	0.08 a	98.7	0.59 a	93.2	0.67 a	95.57
	9.0%	0.00 a	100.0	0.35 a	96.0	0.35 a	97.68
	12.0%	0.00 a	100.0	0.10 a	98.8	0.10 a	99.34
Tetrany	0.0%	7.15 c	0.0	8.90 d	0.0	16.05 d	0.00
	1.0%	5.14 b	28.1	6.94 c	22.0	12.08 c	24.74
	3.0%	0.35 a	95.1	2.69 b	69.8	3.04 b	81.06
	6.0%	0.12 a	98.3	0.79 a	91.1	0.91 a	94.31
	9.0%	0.00 a	100.0	0.65 a	92.7	0.65 a	95.95
	12.0%	0.00 a	100.0	0.15 a	98.3	0.15 a	99.07
Harmoniya	0.0%	4.48 c	0.0	8.05 c	0.0	12.53 c	0.00
	1.0%	3.41 b	23.7	4.73 b	41.2	8.15 b	34.96
	3.0%	0.06 a	98.7	0.60 a	92.6	0.65 a	94.79
	6.0%	0.00 a	100.0	0.50 a	93.8	0.50 a	96.01
	9.0%	0.00 a	100.0	0.10 a	98.8	0.10 a	99.20
	12.0%	0.00 a	100.0	0.05 a	99.4	0.05 a	99.60

**Table 4:** Drought-tolerant indices of the studied ryegrass varieties under field conditions.

Genotype/ Variety	Indices				
	*DTI*	*DSI*	*SSPI*	*TOL*	*YI*
Harmoniya	0.88	0.020	0.214	18.23	0.486
Tetrany	1.69	0.014	0.385	32.77	1.069
Tetramis	3.58	0.037	0.846	72.01	1.445

**Table 5:** Influence of factors on seed germination according to ANOVA of the data obtained. The means showing different letters are significantly different according to Tukey’s post hoc test (*p* < 0.05).

Factor	Variable	Weight
Factor A Genotype	a1	36.25 b
	a2	39.17 b
	a3	24.17 a
Factor B Sucrose concentration, %	b1	81.7 f
	b2	62.5 e
	b3	30.0 d
	b4	14.2 c
	b5	8.3 b
	b6	2.5 a
Factorial relationship	MS	η^2^
A	2277.1	4.4
B	18507.1	90.3
A × B	429.6	4.2

**Table 6:** Influence of factors on plant growth and development according to ANOVA of the data obtained. The means showing different letters are significantly different according to Tukey’s post hoc test (*p* < 0.05).

Factor	Variable	Root		Shoot		Seedling	
Factor A Genotype	a1	1.983 b		2.881 b		4.864 b	
	a2	2.332 b		3.486 c		5.819 c	
	a3	1.044 a		2.239 a		3.282 a	
Factor B Sucrose concentration, %	b1	6.025 c		8.533 d		14.558 d	
	b2	4.410 b		5.903 c		10.312 c	
	b3	0.256 a		1.693 b		1.949 b	
	b4	0.070 a		0.592 a		0.591 a	
	b5	0.000 a		0.368 a		0.357 a	
	b6	0.000 a		0.123 a		0.162 a	
Factorial relationship		MS	η^2^	MS	η^2^	MS	η^2^
A		9.10	1.0	13.09	0.9	43.54	1.0
B		277.41	72.9	455.03	79.9	1438.53	83.4
A × B		4.30	2.3	2.11	0.7	7.26	0.8

**Table 7:** Correlation analysis of the studied ryegrass varieties under laboratory conditions.

Genotype/ Variety	Seed Germination	Length, cm			Seedling Biomass, g
		Root	Shoot	Seedling	
Harmoniya	−0.895	−0.792	−0.844	−0.822	−0.966
Tetrany	−0.927	−0.788	−0.880	−0.842	−0.937
Tetramis	−0.850	−0.774	−0.787	−0.785	−0.835

## Data Availability

The raw data supporting the conclusions of this article will be made available by the authors on request.
